# Independent amplification of co-infected long incubation period low conversion efficiency prion strains

**DOI:** 10.1371/journal.ppat.1007323

**Published:** 2018-10-18

**Authors:** Thomas E. Eckland, Ronald A. Shikiya, Jason C. Bartz

**Affiliations:** Department of Medical Microbiology and Immunology, School of Medicine, Creighton University, Omaha, Nebraska, United States of America; Dartmouth Medical School, USA, UNITED STATES

## Abstract

Prion diseases are caused by a misfolded isoform of the prion protein, PrP^Sc^. Prion strains are hypothesized to be encoded by strain-specific conformations of PrP^Sc^ and prions can interfere with each other when a long-incubation period strain (i.e. blocking strain) inhibits the conversion of a short-incubation period strain (i.e. non-blocking). Prion strain interference influences prion strain dynamics and the emergence of a strain from a mixture; however, it is unknown if two long-incubation period strains can interfere with each other. Here, we show that co-infection of animals with combinations of long-incubation period strains failed to identify evidence of strain interference. To exclude the possibility that this inability of strains to interfere *in vivo* was due to a failure to infect common populations of neurons we used protein misfolding cyclic amplification strain interference (PMCAsi). Consistent with the animal bioassay studies, PMCAsi indicated that both co-infecting strains were amplifying independently, suggesting that the lack of strain interference is not due to a failure to target the same cells but is an inherent property of the strains involved. Importantly PMCA reactions seeded with long incubation-period strains contained relatively higher levels of remaining PrP^C^ compared to reactions seeded with a short-incubation period strain. Mechanistically, we hypothesize the abundance of PrP^C^ is not limiting *in vivo* or *in vitro* resulting in prion strains with relatively low prion conversion efficiency to amplify independently. Overall, this observation changes the paradigm of the interactions of prion strains and has implications for interspecies transmission and emergence of prion strains from a mixture.

## Author summary

This is the first example of prion strains that replicate independently *in vitro* and *in vivo*. This observation changes the paradigm of the interactions of prion strains and provides further evidence that strains are a dynamic mixture of substrains. Strain interference is influenced by the abundance of PrP^C^ that is convertible by the strains involved. These observations have implications for interspecies transmission and emergence of prion strains from a mixture.

## Introduction

Prion diseases are a group of transmissible neurodegenerative diseases that affect animals, including humans. Animal prion diseases include scrapie in sheep and goats, transmissible mink encephalopathy (TME) in ranch-raised mink, chronic wasting disease (CWD) in cervids, and bovine spongiform encephalopathy [1–10]. The human prion diseases can be acquired, inherited, or can occur sporadically and include Creutzfeldt-Jakob disease (CJD), Gerstmann-Straussler-Scheinker disease, fatal familial insomnia, and kuru [11–15]. Prion diseases have long asymptomatic incubation periods ranging from months to decades followed by a short symptomatic phase characterized by progressive cognitive and/or motor deficits [16,17]. During the asymptomatic phase, prions can be detected in the central nervous system and extraneural locations [18]. Currently, effective treatment for prion diseases is not available, and they are inevitably fatal.

The prion agent is comprised mainly, if not entirely, of PrP^Sc^ which is an abnormal isoform of the host encoded prion protein, PrP^C^ [19–23]. Prion propagation is thought to occur in a three-step process where PrP^Sc^ first binds to PrP^C^ followed by a conformational conversion of PrP^C^ to PrP^Sc^. Next, fragmentation of the growing PrP^Sc^ polymer results in the generation of new PrP^Sc^ free ends for PrP^C^ to bind. Repeated cycles of this process are thought to encompass prion formation *in vivo* and are recapitulated *in vitro* by protein misfolding cyclic amplification (PMCA) [24].

Prion strains are operationally defined as a phenotype of disease under a fixed set of agent and host parameters. Under experimental conditions where these parameters are precisely controlled, distinct phenotypes of disease correspond with prion strains [25–27] [28–36]. Differences in the distribution and relative intensity of spongiform degeneration in select areas of the central nervous system (CNS) are, currently, the most well-accepted criteria to distinguish strains [37,38]. Prion strains can differ in incubation period, clinical signs of disease, tissue tropism, and host range. Importantly, the strain specific phenotype is maintained upon serial passage and is, therefore, heritable.

Prion strain diversity may be encoded by distinct conformations of PrP^Sc^. Studies using the hyper (HY) and drowsy (DY) strains of hamster-adapted TME were the first to show that PrP^Sc^ could have strain-specific differences in the proteinase K (PK) cleavage site, with the unglycosylated PrP^Sc^ polypeptide migrating at 21 and 19 kDa respectively, relative PK resistance and detergent insolubility [39–41]. Consistent with these findings, similar strain-specific differences in PrP^Sc^ migration properties of human prion isolates were preserved upon transmission to transgenic mice expressing chimeric mouse-human PrP^C^ [42]. Structural studies of PrP^Sc^ using Fourier transform infrared spectroscopy indicated that strain-specific differences in PrP^Sc^ secondary structure may underlie strain-specific properties of PrP^Sc^ such as PK cleavage site [43,44]. The conformation dependent immunoassay (CDI) measures changes in immunoreactivity of PrP^Sc^ compared to immunoreactivity of PrP^C^ under conditions of increasing denaturation. CDI has identified strain-specific differences in PrP^Sc^ conformation from several rodent prion strains [45]. The relationship between the strain-specific biochemical features of PrP^Sc^ and the outcome of disease are poorly understood.

Prion strains, when present in the same host, can interfere with each other. This was first observed in mice where inoculation of the long incubation period blocking strain, 22C, prior to superinfection with the short incubation period strain,22A, could extend the incubation period of 22A [46]. Short and long incubation period strains are categorized relative to the minimum and maximum incubation periods of known strains for a given host under experimental transmission parameters. As the interval between inoculation of 22C and 22A is increased, 22C can extend the incubation period or completely block 22A from causing disease [46]. This is consistent with subsequent studies indicating that the relative onset of conversion of the blocking and superinfecting strain influences strain emergence, and conversion of the blocking strain PrP^Sc^ corresponds with the occurrence of strain interference [47–49]. Additionally, the blocking and superinfecting strains must infect the same cells for strain interference to occur and potentially compete for PrP^C^ [47,50]. Prion strain interference is a common property of prions and numerous host strain combinations have been identified where prion strains interfere with each other or completely block one strain from causing disease [16,46,51–55]. Most of these studies, however, investigated combinations of prion strains that have large differences in incubation period and/or prion agent conversion efficiencies. It is unknown if two relatively long-incubation period, low efficiency converting prion strains can interfere with each other. In this study we investigated the ability of relatively low prion conversion efficiency strains to interfere with each other *in vivo* and *in vitro* and the contribution of PrP^C^ in this process.

## Results

**Fig 1:**
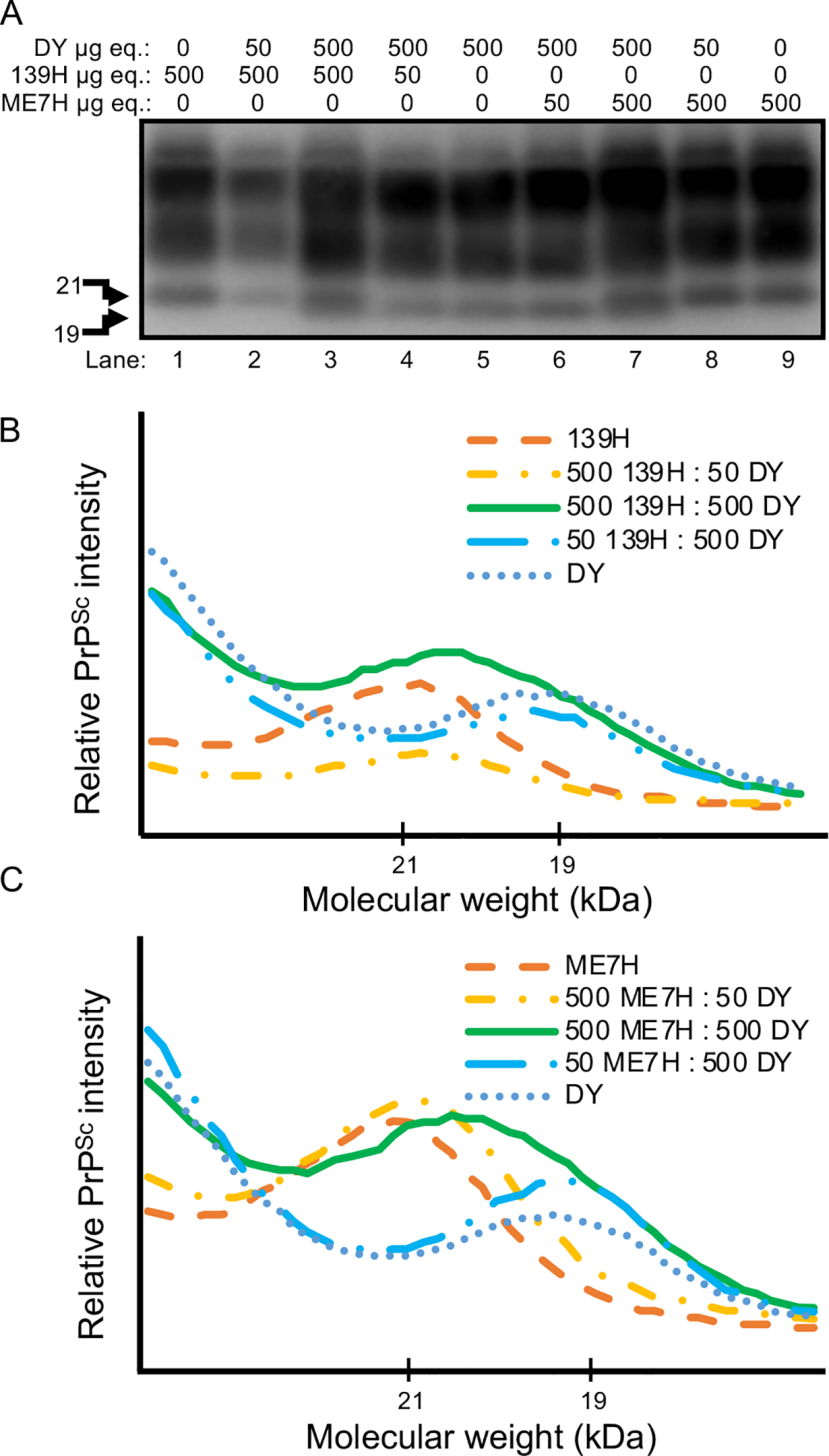
Western blot migration analysis of PrP^Sc^ from 139H, DY and ME7H-infected animals. Western blot (A) and migration analysis (B, C) of PrP^Sc^ from 139H, DY or ME7H-infected PK digested brain homogenates at ratios of 1:10, 1:1, or 10:1. In lanes 1, 2, 8, and 9 the unglycosylated PrP^Sc^ polypeptide migrates at 21 kDa and in lanes 4, 5, and 6 the unglycosylated PrP^Sc^ polypeptide migrates at 19 kDa. In lane 3 and 7 the migration of the unglycosylated PrP^Sc^ polypeptide migrates from 19 kDa and 21 kDa. This experiment was repeated a minimum of three times with similar results.

*Mixtures of DY and 139H or DY and ME7H have differentiable PrP*^*Sc*^ *Western blot migration profiles*. To determine the strain-specific migration of PrP^Sc^ from mixtures of strains, Western blot analysis was performed on mixtures of DY and 139H or DY and ME7H brain homogenates at ratios of 10:1, 1:1 and 1:10 (Fig 1). Samples that contained an excess of one strain had unglycosylated PrP^Sc^ polypeptide migration of the excess strain at either 21 or 19 kDa (Fig 1). Samples that contained an equal ratio of DY and 139H or DY and ME7H resulted in the unglycosylated PrP^Sc^ polypeptide migrating at 21 kDa and 19kDa (Fig 1A) that PrP^Sc^ migration analysis confirms as a band migrating from 21 to 19 kDa (Fig 1B and 1C). Overall, a mixture of DY and 139H or DY and ME7H resulted in a dual unglycosylated PrP^Sc^ polypeptide pattern that was resolved by Western blot if the ratio of the two strains was within 10-fold of each other.

**Fig 2:**
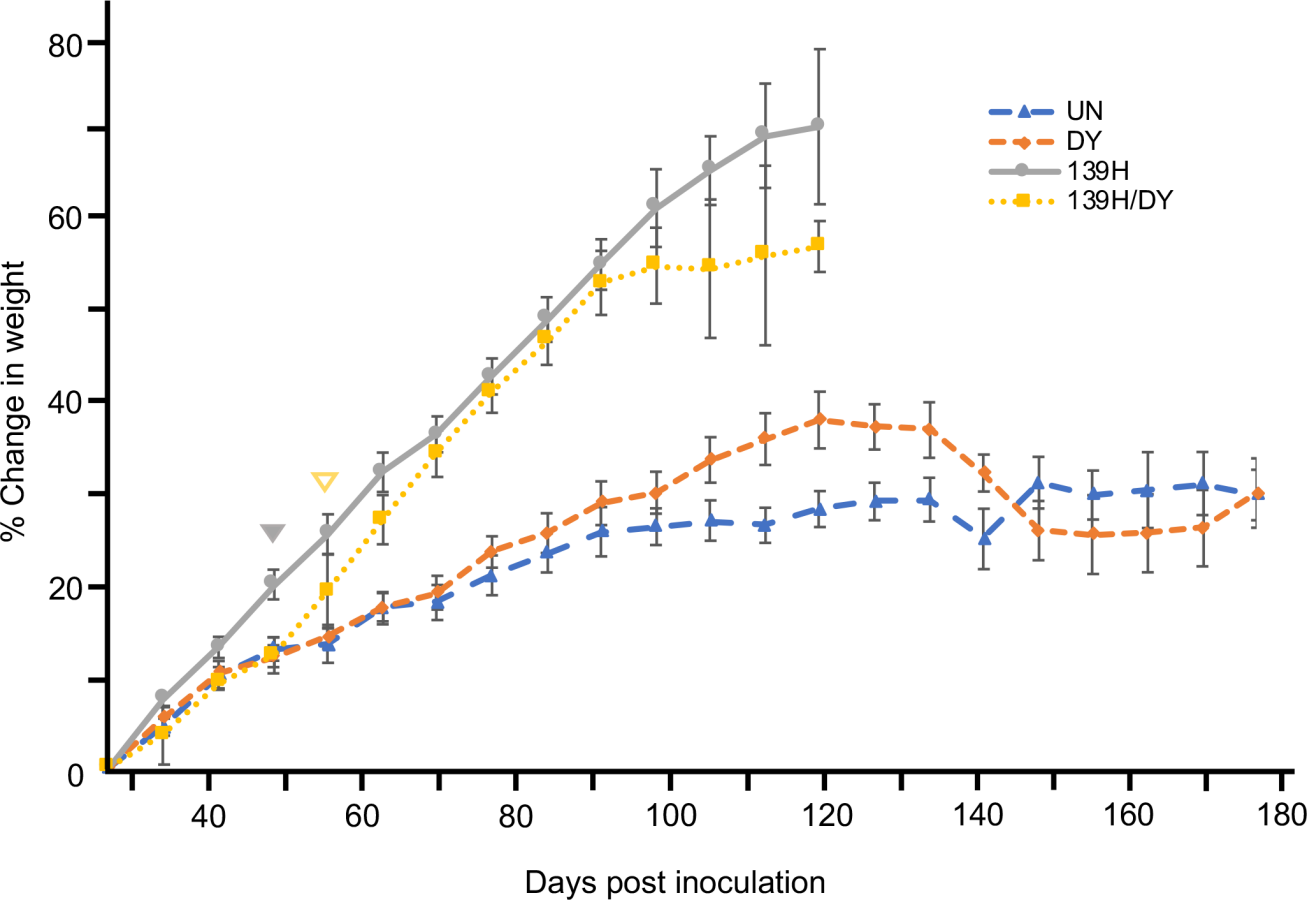
Effect of prion infection on weight gain over the incubation period of disease. Hamsters were inoculated with either uninfected brain homogenate (blue triangles), DY-infected brain homogenate (orange diamonds), 139H-infected brain homogenate (gray circles) or an equal mixture of 139H and DY-infected brain homogenate (yellow squares) and their weights were recorded weekly. Solid grey triangle and open yellow triangle indicates when the weight of 139H-infected or 139H/DY co-infected hamsters, respectively, significantly (p<0.05) differed from mock-infected animals.

**Fig 3:**
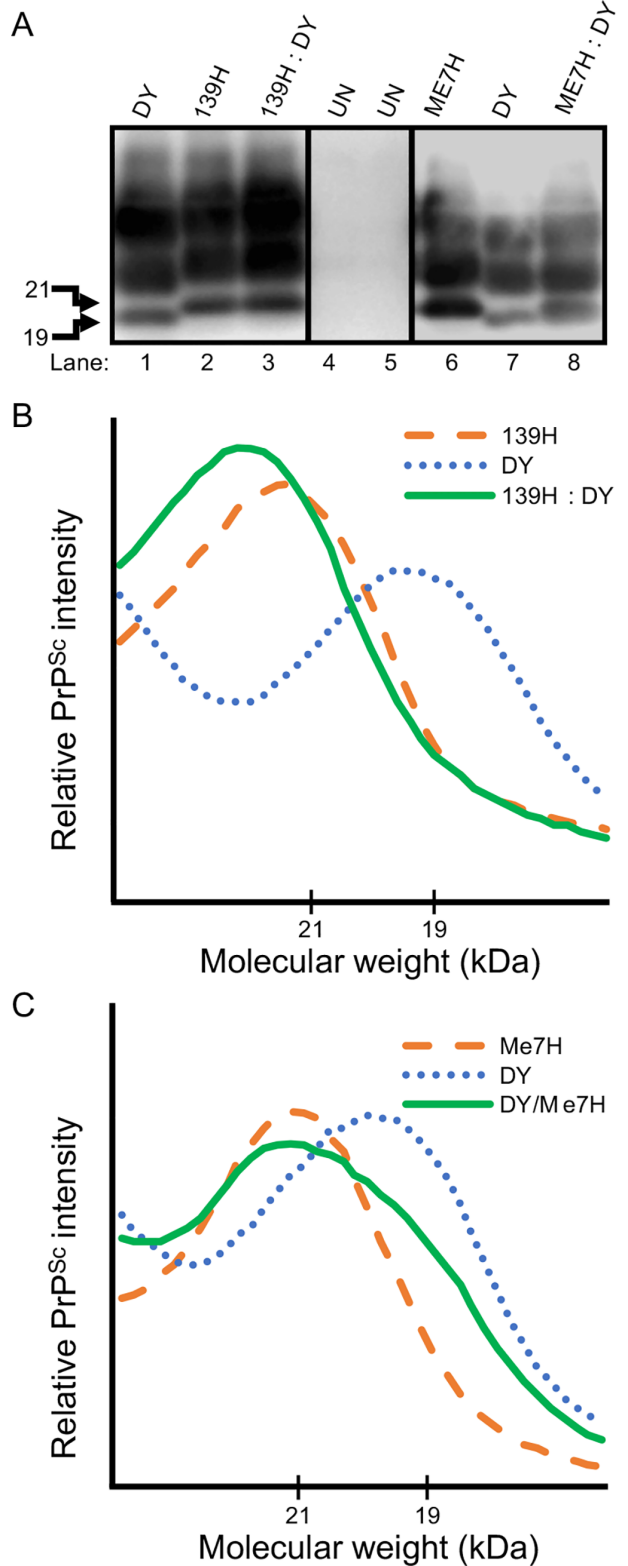
Western blot migration of 139H and DY interference or ME7H and DY interference animal bioassay. Western blot (A) and migration analysis (B, C) of PK digested brain material from hamsters inoculated with either uninfected (UN) brain homogenate, DY-infected brain homogenate, 139H-infected brain homogenate, ME7H-infected brain homogenate or an equal mixture of either DY and 139H or DY and ME7H. In lanes 1 and 7 the unglycosylated PrP^Sc^ polypeptide of the DY positive controls migrates at 19 kDa. In lanes 2, 3, and 6 the unglycosylated PrP^Sc^ polypeptide of the 139H and ME7H-infected positive controls migrates at 21 kDa. In lane 8 the migration of the unglycosylated PrP^Sc^ polypeptide from the animals co-infected with DY and ME7H migrates from 19 to 21 kDa.

*Strain interference does not occur between DY and 139H or DY and ME7H in vivo*. To investigate strain interference *in vivo*, groups of hamsters (n = 5) were intracerebrally (i.c). inoculated with either an uninfected brain homogenate (negative control), DY-infected brain homogenate (positive control), 139H-infected brain homogenate (positive control), ME7H-infected brain homogenate (positive control), or co-infected with an equal ratio of DY and 139H (experimental group 1; Table 1) or DY and ME7H (experimental group 2; Table 1). Positive controls hamsters inoculated with DY (n = 5) all developed clinical signs of progressive lethargy at either 178±4 or 161±3 (Table 1) days p.i. with weight gain that did not significantly (p>0.05) differ from the uninfected negative control animals (Fig 2) and brain material contained an unglycosylated PrP^Sc^ polypeptide that migrated at 19 kDa (Fig 3A, lanes 1 & 7, B and C). All (n = 5) of the 139H-inoculated positive control animals developed clinical signs of ataxia at 125±3 days p.i. (Table 1) with a significant (p<0.05) gain in weight compared to age-matched mock-infected controls starting at 49 days p.i. that continued for the duration of the disease course (Table 1 and Fig 2). Western blot of brain material from the 139H-infected animals contained an unglycosylated PrP^Sc^ polypeptide that migrated at 21 kDa (Fig 3A, lane 2 and B). All of the negative control mock-infected animals (n = 5) remained asymptomatic for either 200 or 270 (Table 1) days post-infection (p.i.) and PrP^Sc^ was not detected in brains from these animals by Western blot (Fig 3A, lanes 4–5; cropped from a different blot performed concurrently with the rest of the figure). Hamsters (n = 5) co-infected with the DY and 139H agents developed clinical signs of ataxia at 125±3 days p.i. that did not significantly (p>0.05) differ from animals inoculated with 139H alone but did differ significantly (p<0.05) from animals inoculated with DY alone (Table 1). The DY and 139H co-infected animals had a statistically significant (p<0.05) weight gain starting at 56 days p.i. compared to age-matched mock infected animals but did not differ significantly (p>0.05) from animals inoculated with 139H (Fig 2). At 119 days p.i. an intercurrent death occurred in both the 139H and the DY/139H co-infected group resulting in a reduction of statistical power that may have contributed to the lack of statistical significance at this time point (Fig 2). Brain material from hamsters co-infected with DY and 139H agents had unglycosylated PrP^Sc^ that migrated at 21 kDa consistent with 139H infection (Fig 3A, lane 3 and B). All (n = 5) of the ME7H-inoculated positive controls developed clinical signs of prion infection at 250±3 days p.i. (Table 1), and Western blot of brain material from the ME7H-infected animals contained an unglycosylated PrP^Sc^ polypeptide that migrated at 21 kDa (Fig 3A, lane 6 and C). Hamsters co-infected with the DY and ME7H developed clinical signs of progressive lethargy at 161±3 days p.i. that did not significantly differ (p>0.05) from animals inoculated with DY alone but did statistically differ (p<0.05) from animals inoculated with ME7H alone (Table 1). Brain material from hamsters co-infected with DY and ME7H agents had unglycosylated PrP^Sc^ that migrated from 19 to 21 kDa consistent with a mixture of both DY and ME7H PrP^Sc^ (Fig 3A, lane 8 and C).

**Table 1:** Co-infection with DY and 139H or ME7H scrapie does not result in strain interference.

Exp.	Inoculum	Incubation period^a^	A/I^b^
1	UN	>200	0/5
	DY	178 ± 4	5/5
	139H	125 ± 3	4/5^c^
	139H/DY	125 ± 3	4/5^c^
2	UN	>270	0/5
	DY	161 ± 3	5/5
	ME7H	250 ± 3	5/5
	ME7H/DY	161 ± 3	5/5

^a^ days ± standard error of the mean to onset of clinical signs

^b^ (number affected / number inoculated)

^c^ one intercurrent death

**Fig 4:**
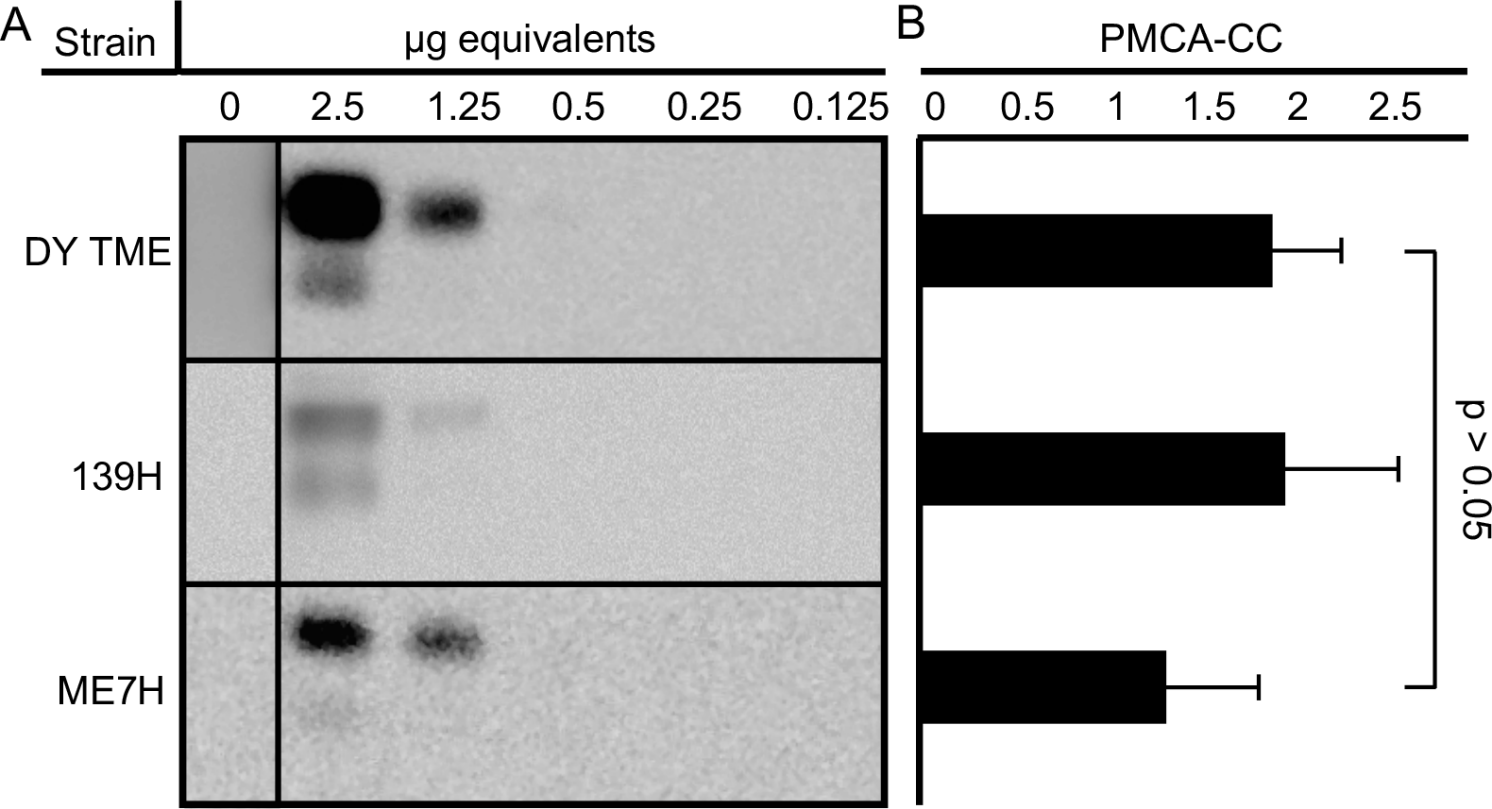
DY, 139H, and ME7H have similar PMCA conversion coefficients. Western blot (A) detection of PrP^Sc^ from PK digested PMCA reactions seeded with serial dilutions of either DY, 139H or ME7H-infected brain homogenate after one round of PMCA. The PMCA-CC of DY, 139H, or ME7H seeded reactions (B) did not significantly (p>0.05) differ. This experiment was repeated a minimum of three times with similar results.

*Similar PMCA conversion efficiency of DY*, *139H*, *and ME7H PrP*^*Sc*^. Previous work indicated the PMCA conversion coefficients (PMCA-CC) of DY, 139H, and ME7H scrapie are similar based on 10-fold serial dilutions of brain homogenate [56]. The PMCA-CC of these strains was further refined by examining selected dilutions of infected brain homogenate that differed by less than 10-fold (Fig 4). The PMCA-CC was 1.80 ± 0.35 for DY, 1.89 ± 0.44 for 139H, and 1.33 ± 0.42 for ME7H. These PMCA-CC values did not differ statistically (p>0.05) when compared to DY. Mock-infected PMCA reactions did not amplify detectable PrP^Sc^ (Fig 4; DY sample was cropped from a separate blot that was performed concurrently)

**Fig 5:**
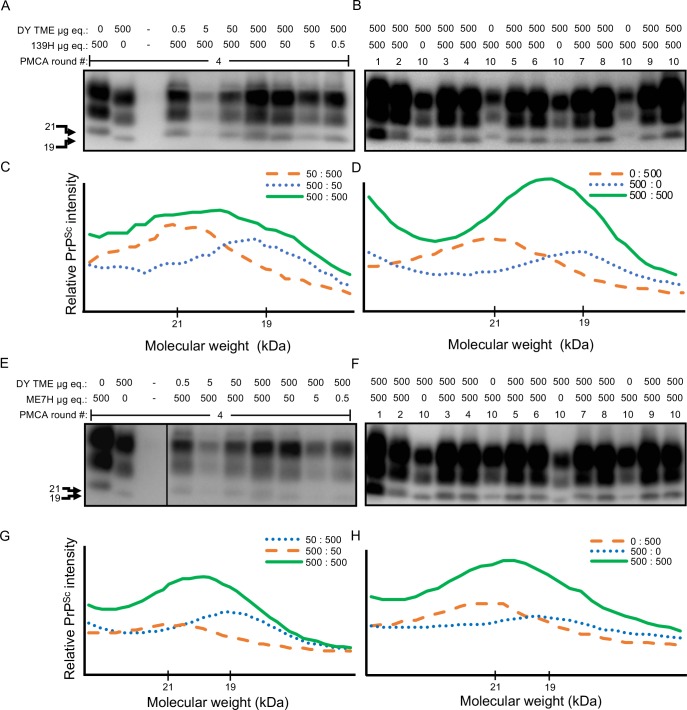
Low conversion efficiency prion strains do not interfere in PMCA. Western blot (panels A, B, E, F) and PrP^Sc^ migration analysis (panels C, D, G, H) of PMCAsi reactions seeded with either DY and 139H (A through D) or DY and ME7H-infected brain homogenate (E through H). Shown is PMCAsi round 4 with the tested ratios of DY and 139H (panels A and C) or DY and ME7H (panels E and G) or PMCAsi rounds 1 through 10 of and equal mixture of DY and 139H (panels B and D) or DY and ME7H (panels F and H). This experiment was repeated a minimum of three times with similar results.

*Lack of strain interference between long-incubation period*, *low efficiency converting prion strains in vitro*. PMCAsi was performed as previously described [50], and all PMCAsi reactions were performed in triplicate. Unseeded PMCA negative control reactions did not result in detectable PrP^Sc^ (Tables 2 and 3). Positive PMCA control reactions seeded with either DY, 139H or ME7H resulted in amplification of strain-specific PrP^Sc^ (Tables 2 and 3; Fig 5). For PMCAsi reactions, known ratios of DY and 139H (Table 2) or DY and ME7H (Table 3) were mixed and subjected to serial rounds of PMCA. The migration of PrP^Sc^ was determined by Western blot using the parameters described in Fig 1. In the co-infected PMCAsi reactions, the migration of PrP^Sc^ was similar to the strain that was initially seeded in excess (Tables 2 and 3; Fig 5). When an equal ratio of strains was used as the starting material we found a 19 to 21 kDa migration of unglycosylated PrP^Sc^ consistent with amplification of both strains that was maintained for 10 serial rounds of PMCA (Tables 2 and 3; Fig 5).

**Table 2:** 139H and DY amplify independently in PMCA.

μg br. eq.		PMCA Round									
139H	DY	1	2	3	4	5	6	7	8	9	10
0	0	-	-	-	-	-	-	-	-	-	-
500	0	139H	139H	139H	139H	139H	139H	139H	139H	139H	139H
50	0	139H	139H	139H	139H	139H	n.d.	n.d.	n.d.	n.d.	n.d.
5	0	139H	139H	139H	139H	139H	n.d.	n.d.	n.d.	n.d.	n.d.
0.5	0	139H	139H	139H	139H	139H	n.d.	n.d.	n.d.	n.d.	n.d.
0	500	DY	DY	DY	DY	DY	DY	DY	DY	DY	DY
0	50	DY	DY	DY	DY	DY	n.d.	n.d.	n.d.	n.d.	n.d.
0	5	DY	DY	DY	DY	DY	n.d.	n.d.	n.d.	n.d.	n.d.
0	0.5	DY	DY	DY	DY	DY	n.d.	n.d.	n.d.	n.d.	n.d.
0.5	500	DY	DY	DY	DY	DY	DY	DY	DY	DY	DY
5	500	DY	DY	DY	DY	DY	DY	DY	DY	DY	DY
50	500	DY	DY	DY	DY	DY	DY	DY	DY	DY	DY
500	500	Mix	Mix	Mix	Mix	Mix	Mix	Mix	Mix	Mix	Mix
500	50	139H	139H	139H	139H	139H	139H	139H	139H	139H	139H
500	5	139H	139H	139H	139H	139H	139H	139H	139H	139H	139H
500	0.5	139H	139H	139H	139H	139H	139H	139H	139H	139H	139H

Br.–brain, eq., equivalents; DY, PrP^Sc^ with DY migration pattern; 139H, PrP^Sc^ with 139H migration pattern;–, PrP^Sc^ not detected; n.d., not done.

**Table 3:** Lack of strain interference between DY and ME7H scrapie in PMCA.

μg br. eq.		PMCA Round									
ME7H	DY	1	2	3	4	5	6	7	8	9	10
0	0	-	-	-	-	-	-	-	-	-	-
500	0	ME7H	ME7H	ME7H	ME7H	ME7H	ME7H	ME7H	ME7H	ME7H	ME7H
50	0	ME7H	ME7H	n.d.	n.d.	n.d.	n.d.	n.d.	n.d.	n.d.	n.d.
5	0	ME7H	ME7H	n.d.	n.d.	n.d.	n.d.	n.d.	n.d.	n.d.	n.d.
0.5	0	ME7H	ME7H	n.d.	n.d.	n.d.	n.d.	n.d.	n.d.	n.d.	n.d.
0	500	DY	DY	DY	DY	DY	DY	DY	DY	DY	DY
0	50	DY	DY	n.d.	n.d.	n.d.	n.d.	n.d.	n.d.	n.d.	n.d.
0	5	DY	DY	n.d.	n.d.	n.d.	n.d.	n.d.	n.d.	n.d.	n.d.
0	0.5	DY	DY	n.d.	n.d.	n.d.	n.d.	n.d.	n.d.	n.d.	n.d.
0.5	500	DY	DY	DY	DY	DY	DY	DY	DY	DY	DY
5	500	DY	DY	DY	DY	DY	DY	DY	DY	DY	DY
50	500	DY	DY	DY	DY	DY	DY	DY	DY	DY	DY
500	500	Mix	Mix	Mix	Mix	Mix	Mix	Mix	Mix	Mix	Mix
500	50	ME7H	ME7H	ME7H	ME7H	ME7H	ME7H	ME7H	ME7H	ME7H	ME7H
500	5	ME7H	ME7H	ME7H	ME7H	ME7H	ME7H	ME7H	ME7H	ME7H	ME7H
500	0.5	ME7H	ME7H	ME7H	ME7H	ME7H	ME7H	ME7H	ME7H	ME7H	ME7H

Br.–brain, eq., equivalents; DY, PrP^Sc^ with DY migration pattern; ME7H, PrP^Sc^ with ME7H migration pattern;–, PrP^Sc^ not detected; n.d., not done.

**Fig 6:**
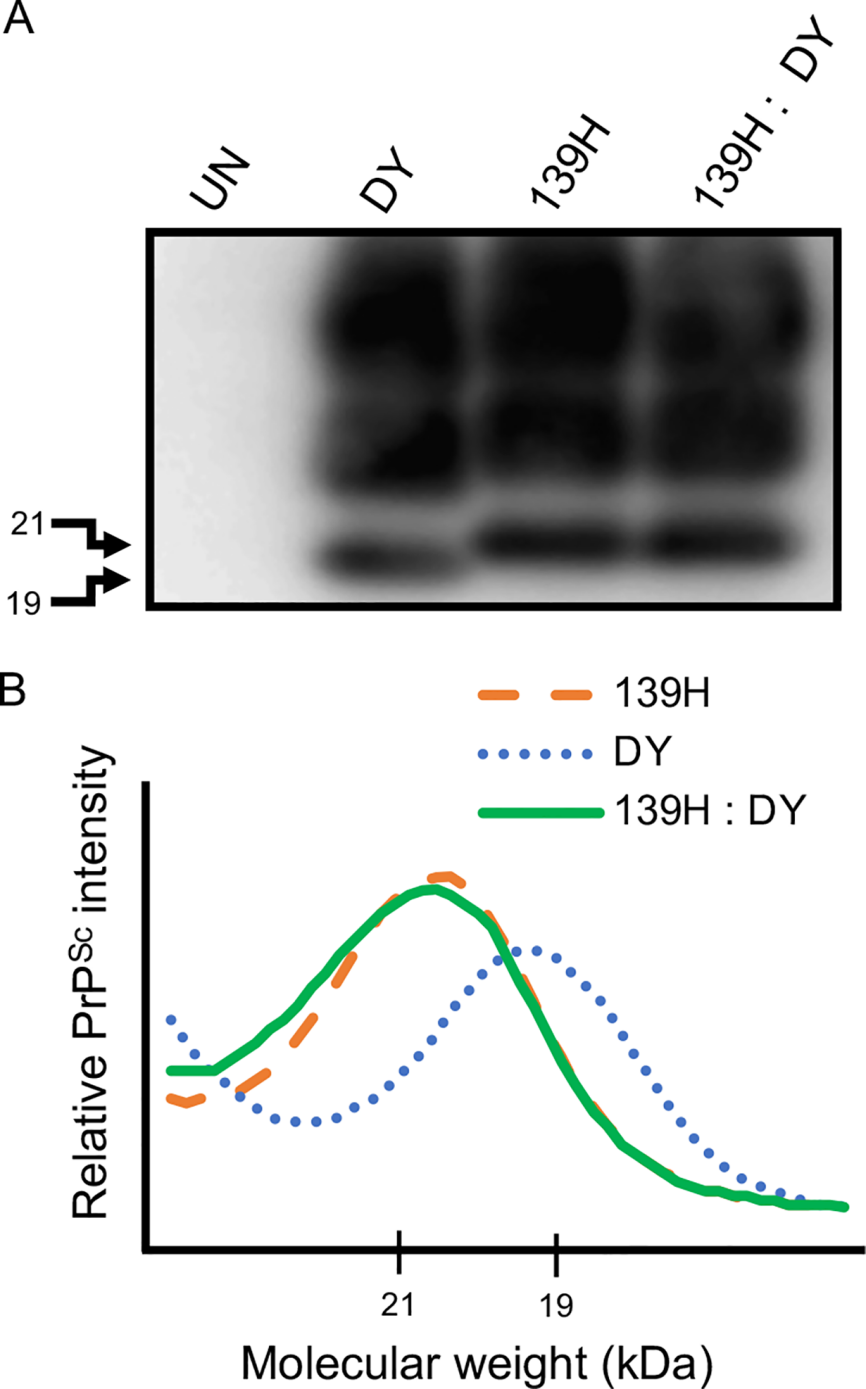
Preservation of strain specific PrP^Sc^ migration following PMCA. Western blot analysis (A) and PrP^Sc^ migration analysis (B) of PK digested brain material from hamsters inoculated with either 10^th^ serial round PMCA reactions seeded with uninfected (UN) brain homogenate, DY, 139H or an equal mixture of DY and 139H-infected brain homogenate.

*PMCAsi did not generate a new prion strain*. PMCAsi reactions seeded with equal amounts of the long incubation period strains contain unglycosylated PrP^Sc^ that migrates from 19 to 21 kDa. This PrP^Sc^ species may not represent independent amplification of each strain but instead represent a new prion strain. To test this hypothesis, groups (n = 5) of hamsters were i.c. inoculated with round 10 PMCAsi material from either uninfected negative control PMCA reactions, DY or 139H seeded positive control PMCA reactions or reactions seeded with equal amounts of DY and 139H. The hamsters inoculated with the uninfected negative control PMCA samples remained asymptomatic for 275 p.i., weighed 152.4±5.0 g at 172 p.i., (Table 4) and PrP^Sc^ was not detected in brain material from these animals (Fig 6A). Hamsters inoculated with the round 10 PMCA DY positive control reaction developed clinical signs of progressive lethargy at 224±7 days p.i., weighed 170.6±5.9 g at 172 p.i. (Table 4), and brain material from all animals contained an unglycosylated PrP^Sc^ polypeptide that migrated at 19 kDa consistent with DY infection (Fig 6). Hamsters inoculated with the round 10 PMCA 139H seeded positive control reactions developed clinical signs of ataxia at 177±3 days p.i., weighed 203.4± 6.0 g at 172 p.i. (Table 4), and brain material from all animals contained an unglycosylated PrP^Sc^ polypeptide that migrated at 21 kDa consistent with 139H infection (Fig 6). Hamsters inoculated with round 10 PMCAsi DY and 139H seeded experimental group developed clinical signs of ataxia at 180±3 days p.i., weighed 198.0±5.5 g (Table 4) and brain material all of these animals contained an unglycosylated PrP^Sc^ polypeptide that migrated at 21 kDa consistent with 139H infection (Fig 6). The incubation period of animals inoculated with round 10 PMCA 139H and round 10 PMCAsi mixture of DY and 139H did not differ significantly (p>0.05) but did differ significantly (p<0.05) from animals inoculated with either round 10 PMCA DY or UN PMCA samples (Table 4). The weight of hamsters at the onset of clinical disease inoculated with round 10 PMCAsi DY and 139H mixture did not statistically (p>0.05) differ from the weights of hamsters inoculated with round 10 PMCA 139H reactions but did differ statistically (p<0.05) from animals inoculated with round 10 PMCA DY or UN PMCA reactions (Table 4). Overall, the hamsters inoculated with the round 10 PMCAsi DY and 139H mixture had the same incubation period, clinical signs, weight gain, and PrP^Sc^ properties of the round 10 PMCAsi 139H group. This is consistent with our findings that 139H and DY do not interfere (Tables 1 and 2) and suggest that a new prion strain was not produced.

**Table 4:** Infectivity of PMCA generated DY and 139H prions.

Inoculum	Incubation period^a^	A/I^b^	Weight (grams)^c^
UN	>275	0/5	152.4 ± 5.0
DY	224 ± 7	5/5	170.6 ± 5.9
139H	177 ± 3	5/5	203.4 ± 6.0
139H/DY	180 ± 3	5/5	198.0 ± 5.5

^a^ days ± standard error of the mean to onset of clinical signs

^b^ (number affected / number inoculated)

^c^ at onset of clinical disease

**Fig 7:**
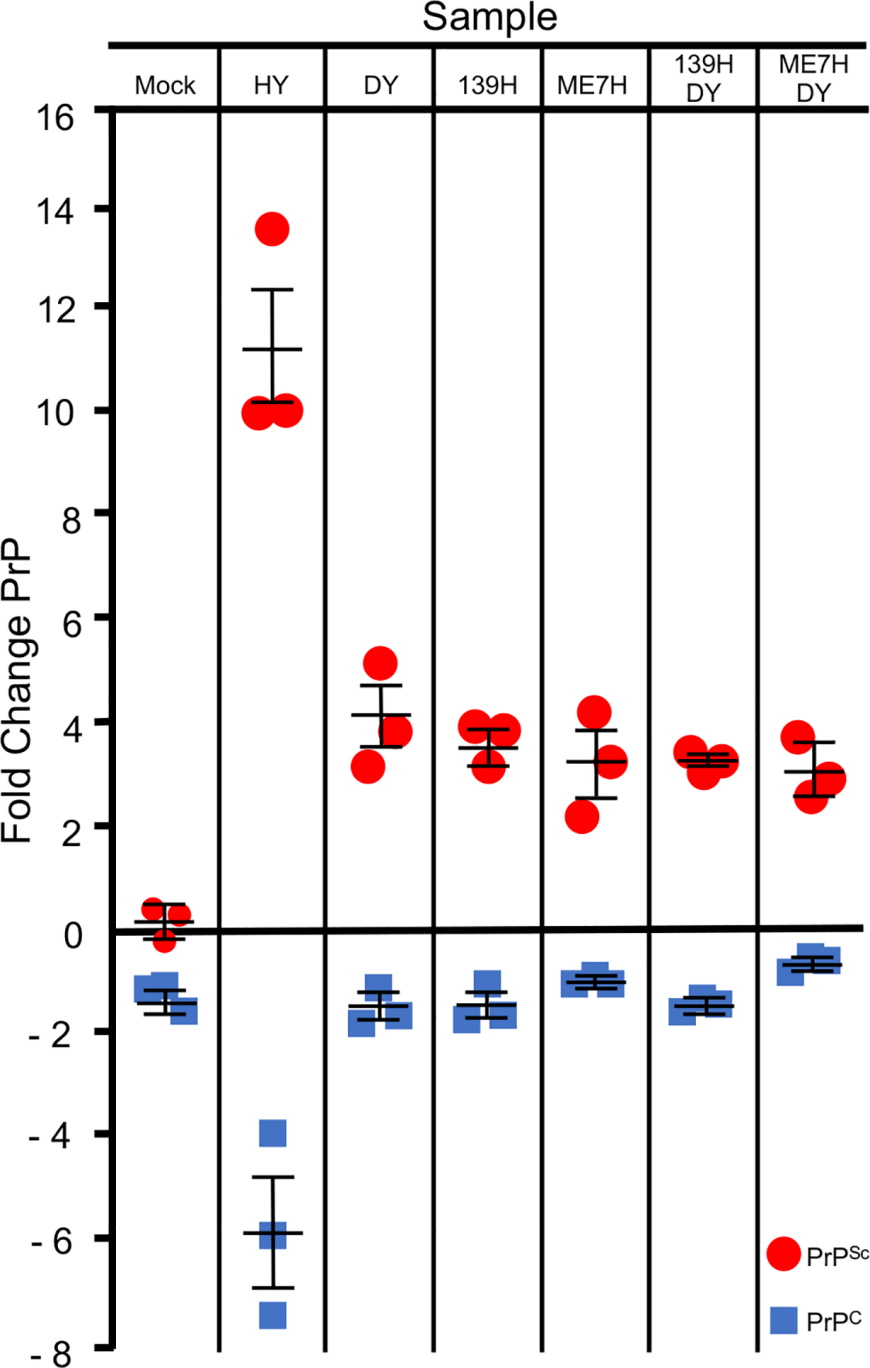
PrP^C^ abundance is not limiting for low conversion efficiency prion strains in PMCAsi. Epitope accessibility immunoassay measurement of the fold change of PrP^C^ (blue squares) and PrP^Sc^ (red circles) after one round of PMCA or PMCAsi from reactions seeded with either uninfected (UN), HY-infected, DY-infected, 139H-infected, ME7H-infected, or co-infected with DY and 139H scrapie-infected, or DY and ME7H-infected brain homogenates. Error bars represent SEM. The experiment was repeated a minimum of three times with similar results.

*Abundance of PrP*^*C*^ *and PrP*^*Sc*^ *in PMCA strain interference reactions*. The relative amounts of PrP^C^ and PrP^Sc^ in a single PMCA reaction can be determined using the epitope accessibility immunoassay (EAI) (Fig 7). In the mock-infected seeded reactions, PrP^C^ abundance was significantly (p <0.05) reduced (1.32±0.06 fold), and PrP^Sc^ abundance was not detected (0.06±0.21) compared to the unamplified control (Fig 7). In reactions seeded with HY, a positive control short incubation period high efficiency PrP^Sc^ converting strain, PrP^C^ abundance was significantly (p<0.05) reduced (5.86±1.04 fold) and PrP^Sc^ abundance significantly (p<0.05) increased (11.16±1.01 fold) compared to the unamplified control (Fig 7). In reactions seeded with either DY, 139H or ME7H, PrP^C^ abundance was significantly (p<0.05) reduced (1.62±0.13, 1.58±0.16 or 1.12±0.04 fold) and PrP^Sc^ abundance significantly (p<0.05) increased (4.02±0.61, 3.47±0.22 or 3.31±0.62 fold) respectively compared to the unamplified control (Fig 7). In reactions seeded with either 139H and DY, or ME7H and DY, PrP^C^ abundance was significantly (p<0.05) reduced (1.63±0.07 or 0.81±0.07 fold) and PrP^Sc^ abundance significantly (p<0.05) increased (3.32±0.13 or 3.11±0.36 fold) respectively compared to the unamplified control (Fig 7). A significant (p<0.05) fold decrease of PrP^C^ abundance was found for HY compared to DY, 139H, ME7H, 139H / DY mixture, and ME7H / DY mixture samples. A significantly (p<0.05) greater fold increase of PrP^Sc^ abundance was found for HY when compared to DY, 139H, ME7H, 139H / DY mixture, and ME7H / DY mixture (Fig 7). Overall, we found that prion strains with a relatively lower prion conversion efficiency consume correspondingly lower amounts of PrP^C^, compared to HY that has a relatively higher efficiency of prion conversion.

## Discussion

Previous strain interference studies have examined the capacity of a long-incubation period strain that have low prion conversion efficiencies to interfere with a short-incubation strains that have relatively higher prion conversion efficiencies [46,51–55]. To investigate if two long-incubation period low conversion efficiency prion strains can interfere with each other we co-infected hamsters with DY and 139H or ME7H. These three strains have similar relatively low prion conversion efficiencies (Fig 4). Additionally, 139H and ME7H were chosen because 139H has a shorter incubation period compared to DY and the incubation period of ME7H is much longer. In addition, all three of these strains have a longer incubation period compared with short-incubation period hamster strains such as HY. We found that co-infection of hamsters with DY in combination with either 139H or ME7H resulted in animals developing clinical signs of disease with an incubation period comparable to animals inoculated with the shorter incubation period strain alone (Table 1), suggesting that strain interference was not occurring. Additionally, in the animals co-infected with DY and ME7H, we found evidence that the PrP^Sc^ in these animals contained a mixture of DY and ME7H PrP^Sc^ (Fig 3), providing evidence of independent strain amplification. Based on these *in vivo* experiments, however, we cannot exclude the possibility that DY and ME7H are not infecting the same population of neurons that is needed for strain interference to occur [47]. For example, i.c. inoculation of hamsters with 139H scrapie prior to superinfection with Sc237 scrapie results in animals developing clinical signs, pathology and an incubation period similar to animals inoculated with Sc237 alone [57]. This data suggests that 139H is unable to interfere with Sc237 prions. Recent work using the sciatic nerve route of inoculation (i.sc.) indicates that 139H can block HY, a strain that is similar to Sc237, from causing disease [58]. HY and 139H initially replicate in ventral motor neurons (VMNs), following i.sc. inoculation, suggesting that the previous report of 139H and Sc237 failing to interfere with each other was not an inherent property of the strains, but rather was due to a failure to target both strains to the same location of prion conversion. We were unable to perform strain interference super-infection studies with these combinations of strains using i.sc. inoculation due to constraints on the relationship of incubation period of disease, the relative onset of PrP^Sc^ formation in VMNs and the lifespan of the host. To overcome this obstacle, we used PMCA to further examine strain interference between these long incubation period, low conversion efficiency prion strains since the relative onset of prion conversion of the strains governs which strain will emerge, independent if the strains were co-infected or super-infected [47].

Low prion conversion efficiency strains amplify independently when present together in PMCA. Strain interference can be recapitulated with PMCA similar to what is observed *in vivo* [50]. Since both prion strains are present in the same PMCA reaction, this *in vitro* system mimics when both strains are infecting the same cell *in vivo*. We found that in PMCAsi the strain that started in greater abundance maintained its strain properties through all 10 rounds of serial PMCAsi, suggesting the other strain was not able to interfere (Tables 2 and 3; Fig 5). Importantly, we found that when both strains started at an equal ratio, a mixture of the 19 and 21 kDa migrating unglycosylated PrP^Sc^ polypeptide was maintained for 10 rounds of PMCAsi (Tables 2 and 3; Fig 5). Based on the limitations of Western blot to resolve a mixture of PrP^Sc^ from both strains (Fig 1) and the strains used have similar PMCA-CC values (Fig 4) and a new prion strain is not generated (Table 4, Fig 6) we conclude that the strains are amplifying independently. This suggests that the failure of these strain combinations to interfere with each other *in vivo* is not due to a failure to target the same cell, but instead is an inherent property of the strain combinations. The concept that not all prion strain combinations result in interference suggests that strain diversity in naturally-infected animals may be greater than previously thought.

Prion strain interference occurs when PrP^C^ abundance is limiting. Prion formation is dependent on PrP^C^, and evidence suggests that prion strains compete for PrP^C^ [50,59]. By definition, therefore, if the amount of available PrP^C^ for prion conversion is limiting, strain interference will occur. We hypothesized that PrP^C^ abundance was not limiting with the long-incubation period, low prion conversion efficiency strains used in this study. To investigate this hypothesis, we used EAI to measure the PK resistant fraction of PrP and the fraction of PrP that binds to the monoclonal anti-PrP antibody 3F4 in the native state, which we interpret as PrP^C^ (Fig 7). We cannot exclude the possibility that other forms of PrP (e.g. PK sensitive PrP^Sc^) that bind to 3F4 in the native state, in addition to PrP^C^, contribute to the observed results, however, previous reports suggest this is unlikely [60,61]. Using this assay, we determined the abundance of PrP^C^ and PrP^Sc^ in the PMCA reactions before and after amplification, and we found that PrP^C^ levels following one round of PMCA were higher in reactions seeded with either DY, 139H or ME7H compared to PMCA reactions seeded with the relatively higher prion conversion efficiency strain HY (Fig 7). This is consistent with the observation that DY, 139H and ME7H have lower amounts of PrP^Sc^ produced after one round of PMCA and lower PMCA-CC (i.e. lower prion conversion efficiencies) compared to HY (Figs 4 and 7). When two low prion conversion efficiency strains are present in PMCA, after one round of PMCA, the level of PrP^C^ is significantly higher compared to reactions seeded with HY (Fig 7). Overall, we conclude that the PrP^C^ requirements of DY, 139H and ME7H are sufficiently low as to not result in PrP^C^ abundance becoming limiting under the conditions tested; therefore, strain interference does not occur.

Not all forms of PrP^C^ are equally suitable templates for prion conversion. Recent work suggests that the sialylation state of PrP^C^ N-linked glycosylation can influence prion formation. Importantly, prion strains may convert a subset of PrP^C^, based on sialylation, more efficiently than other forms of PrP^C^ [62–64]. Based on these findings, it is possible that the subset of PrP^C^ that is convertible by both strains may have a greater influence on prion strain interference than the total PrP^C^ abundance would suggest. In addition to post-translational modifications of PrP^C^, tissue specific ratios of the C1 and C2 cleavage fragments of PrP^C^ may affect the abundance of convertible PrP^C^ [65–67] and can affect strain emergence.

Prion strain interference can affect the emergence of a strain from a mixture. Interspecies transmission can generate new prion strains that, following intraspecies transmission, result in the emergence of a dominant strain [16,68–73]. Interference between prion strains that compete for limiting PrP^C^ is thought to be an important parameter affecting this process [73]. Consistent with this observation, overexpression of PrP^C^ can lead to the emergence of prion strains that are not identified when PrP^C^ is expressed at physiological levels [74]. Additionally, extraneural prion inoculation can result in the emergence of novel strains, further suggesting that changes in the population of PrP^C^ that is initially infected can result in profound differences in prion strain emergence [75]. This finding is consistent with previous work indicating that the initial population of cells infected by a mixture of strains has a large effect on prion strain emergence [47]. Overall, we hypothesize that the availability of strain-specific convertible PrP^C^ can influence strain interference and alter the emergence of a strain from a mixture.

## Materials and methods

### Ethics statement

All procedures involving animals were approved and in compliance with the Guide for the Care and Use of Laboratory Animals (protocol numbers 811 and 880) by the Creighton University Institutional Animal Care and Use Committee.

### Prion strains

Brains from terminally-ill hamsters inoculated with either the 139H (10^8.1^ i.c. LD_50_/g), ME7H, HY (10^9.3^ i.c. LD_50_/g) or DY (10^7.4^ i.c. LD_50_/g) were homogenized to 10% w/v in Dulbecco’s phosphate buffered saline (DPBS) (Mediatech, Herndon, VA) or in PMCA conversion buffer (phosphate-buffered saline [pH 7.4] containing 5 mM EDTA, 1% [vol/vol] Triton X-100, and Complete protease inhibitor tablet [Roche Diagnostics, Mannheim, Germany] [56]. Uninfected hamster brain was homogenized to 10% w/v in PMCA conversion buffer. All homogenates were stored at -80°C.

### Animal bioassay

Male Syrian hamsters (Harlan-Sprague-Dawley, Indianapolis, IN) were intracerebrally (i.c.) inoculated with 25 μl of an equal mixture of 1% w/v uninfected brain homogenate with either 1% w/v of 139H, ME7H, DY, or equal mixtures of a 1% w/v of DY and 139H or DY and ME7H-infected brain homogenates. PMCAsi generated uninfected brain homogenate, DY, 139H, or DY and 139H mixed material was diluted 1:100 and 25 μl was i.c. inoculated into hamsters. Hamsters were observed three times per week for the onset of clinical signs of prion disease and the incubation period was calculated as the number of days between inoculation and onset of clinical signs. Two tail Student’s T test (Prism Version 4.03, for windows; GraphPad Software Inc., La Jolla, CA) with a p value of 0.01 was used to compare incubation periods [55]. Animals were weighed once per week until the onset of clinical disease and weights were compared using ANCOVA analysis (Prism Version 4.03, for windows; GraphPad Software Inc., La Jolla, CA) with a p value of 0.05. Animals were sacrificed by CO_2_ asphyxiation and brain tissue were collected, flash frozen and stored at -80°C.

### Protein misfolding cyclic amplification

Protein misfolding cyclic amplification strain interference (PMCAsi) was adapted from a previously described protocol [50]. Briefly, samples (n = 3 per group) were placed in a Misonix Q700 sonicator (Farmingdale, NY). The sonicator output was set at amplitude 16 with an average output of 165W during each sonication cycle. The ratio of sonicated sample to uninfected brain homogenate was 1:20 for the reactions with either DY, 139H, or Me7H seeded reactions alone. For reactions with mixtures of 139H and DY or Me7H and DY seeded reactions, the ratio of sonicated to uninfected brain homogenate was 1:20 for the first round, 1:10 for the second round, and 1:2 for the remaining rounds of PMCA. Samples containing uninfected brain homogenate in conversion buffer were included in every round of PMCA as a negative control. The PMCA conversion coefficient is calculated as the reciprocal of the concentration of the highest dilution of prion-infected brain homogenate that resulted in detectable amplified PrP^Sc^ by Western blot following one round of PMCA [56]. PMCA conversion coefficients were compared using a two-tailed Student’s T test (Prism Version 4.03, for windows; GraphPad Software Inc., La Jolla, CA) with a p value of 0.05.

### SDS-PAGE and Western blot analysis

Western blot analysis was performed as previously described [76]. Briefly, samples were digested with 100 U/ml of proteinase K (PK) at 37°C for 30 minutes with constant agitation (Roche Diagnostics Corporation, Indianapolis, IN). The PK digestion was terminated by incubating the samples at 100°C for 10 minutes in gel loading buffer (4% w/v SDS, 2% v/v β- mercapto ethanol, 40% v/v glycerol, 0.004% w/v Bromophenol blue, and 0.5 M Tris buffer pH 6.8). Following size fractionation on 4–12% Bis-Tris gel, the proteins were transferred to immobilon P and Western blot analysis were performed using the anti-PrP antibody 3F4 (final concentration of 0.1 μg/ml; Chemicon; Billerica, MA) to recognize hamster prion protein. The Western blot was developed with Pierce supersignal west femto maximum sensitivity substrate according to manufacturer’s instructions (Pierce, Rockford, IL) and imaged on a Kodak 4000R imaging station (Kodak, Rochester, NY). The abundance and migration of PK resistant PrP^Sc^ was determined using the Kodak molecular imaging software v.5.0.1.27 (New Haven, CT). Cropped images are indicated by a vertical line and are from the same exposure of the same blot unless otherwise noted. The signal intensity of the unglycosylated PrP^Sc^ polypeptide as a function of migration distance was determined using the Kodak molecular imaging software v.5.0.1.27 (New Haven, CT).

### Epitope accessibility immunoassay

The epitope of the monoclonal anti-PrP antibody 3F4 is accessible on PrP^C^ in both native and denatured forms, and is largely unavailable in the native conformation of PrP^Sc^ that can be revealed following denaturation [61,77]. Using combinations of PK digestion and denaturation we investigated the relative abundance of various forms of PrP. Samples first digested with PK and then denatured are defined as PrP^Sc^. Samples that have not been digested with PK or denatured are defined as PrP^C^. To accomplish this, samples were mixed with equal volumes of Dulbecco’s phosphate buffered saline (DPBS) or with PK (2.31 units/mL) and incubated at 37°C for 1 hour. The samples were examined for PrP as described previously with the following modifications [78]. A 96 well plate (Millipore, Billerica, MA) was activated with 150 μL methanol and then washed five times with 150 μL of tween tris buffered saline (TTBS) and centrifuged at 470 x *g* for 30 seconds adding 150 μL of TTBS after the first centrifugation. The samples were diluted into DPBS to a total volume of 150 μl and loaded onto the activated 96 well plate and centrifuged three times at 470 x *g* for 30 seconds adding 150 μl DPBS after the first centrifugation. The plate was incubated with to 0.3% H_2_O_2_ in MeOH for 20 mins and then centrifuged at 470 x *g* for 30 seconds two times adding 150 μL of TTBS after the first centrifugation. Next, one non-PK and one PK digested replicate was incubated with either DPBS or 3M guanidine thiocyanate (Sigma Aldrich, St. Louis, MO) for 10 mins and washed five times with 150 μL of TTBS and centrifuged at 470 x *g* for 30 seconds two times, adding 150 μL of TTBS after the first centrifugation. The wells were incubated with 5% w/v blotto in TTBS for 30 mins at 37°C. Blotto was removed and the 96 well was incubated for 1 hour at 37°C with mouse anti-hamster PrP antibody 3F4 (final concentration of 0.1 μg/ml; Chemicon; Billerica, MA) to recognize hamster prion protein and washed five times with 150 μL of TTBS and centrifuged at 470 x *g* for 30 seconds two times, adding 150 μL of TTBS after the first centrifugation. Next the wells were incubated with the secondary antibody HRP-conjugated goat anti-mouse antibody for 30 mins at 37°C (final concentration of 0.1 μg/ml; Thermo Scientific; Rockford, IL.) and washed five times with 150 μL of TTBS and centrifuged at 470 x *g* for 30 seconds rinsing with 150 μL of TTBS two times adding 150 μL of TTBS after the first centrifugation. The 96 well was developed with 40 μL per well of Pierce Supersignal West Femto Maximum Sensitivity Substrate according to manufacturer’s instructions (Pierce, Rockford, IL) and imaged on a Kodak 4000R Imaging Station. The abundance of PrP was determined using the Kodak molecular imaging software v.5.0.1.27 (New Haven, CT) and a two-tailed Student’s T test (Prism Version 4.03, for windows; GraphPad Software Inc., La Jolla, CA) with a p value of 0.05 was used to compare EAI values.
